# Theaflavin-3,3′-Digallate Suppresses Biofilm Formation, Acid Production, and Acid Tolerance in *Streptococcus mutans* by Targeting Virulence Factors

**DOI:** 10.3389/fmicb.2019.01705

**Published:** 2019-07-26

**Authors:** Sa Wang, Yuan Wang, Ying Wang, Zhuhui Duan, Zongxin Ling, Wenzhi Wu, Suman Tong, Huiming Wang, Shuli Deng

**Affiliations:** ^1^Affiliated Hospital of Stomatology, College of Medicine, Zhejiang University, Hangzhou, China; ^2^State Key Laboratory for Diagnosis and Treatment of Infectious Diseases, Collaborative Innovation Center for Diagnosis and Treatment of Infectious Diseases, The First Affiliated Hospital, College of Medicine, Zhejiang University, Hangzhou, China

**Keywords:** *Streptococcus mutans*, theaflavin-3, 3′-digallate, biofilms, extracellular polymeric substances, extracellular DNA, glucans

## Abstract

As one of the most important cariogenic pathogens, *Streptococcus mutans* has strong abilities to form biofilms, produce acid and tolerate acid. In present study, we found that theaflavin-3,3′-digallate (TF3) had an inhibitory effect on *S. mutans* UA159 *in vitro*. Visualized by field emission-scanning electron microscopy, the suppressed formation of *S. mutans* biofilms grown with TF3 at sub-inhibitory concentrations could be attributed to the reduced biofilm matrix, which was proven to contain glucans and extracellular DNA (eDNA). Glucan-reduced effect of TF3 was achieved by down-regulating expression levels of *gtfB, gtfC*, and *gtfD* encoding glucosyltransferases. Besides, TF3 reduced eDNA formation of *S. mutans* by negatively regulating *lrgA, lrgB*, and *srtA*, which govern cell autolysis and membrane vesicle components. Furthermore, TF3 also played vital roles in antagonizing preformed biofilms of *S. mutans*. Bactericidal effects of TF3 became significant when its concentrations increased more than twofold of minimum inhibitory concentration (MIC). Moreover, the capacities of *S. mutans* biofilms to produce acid and tolerate acid were significantly weakened by TF3 at MIC. Based on real-time PCR (RT-PCR) analysis, the mechanistic effects of TF3 were speculated to comprise the inhibition of enolase, lactate dehydrogenase, F-type ATPase and the agmatine deiminase system. Moreover, TF3 has been found to downregulate LytST, VicRK, and ComDE two component systems in *S. mutans*, which play critical roles in the regulatory network of virulence factors. Our present study found that TF3 could suppress the formation and cariogenic capacities of *S. mutans* biofilms, which will provide new strategies for anti-caries in the future.

## 1. Introduction

Dental caries, a deleterious disease, affects more than 35% of people at all ages globally (Pitts et al., 2017), but effective strategies to prevent and manage this disease are still needed. Caries is related to a high-carbohydrate diet, host susceptibility, cariogenic dental plaques, and other factors (Pitts et al., 2017). Dental plaques, the pivotal pathogenic factor, consist of bacteria and extracellular polymeric substances (EPSs) (Hajishengallis et al., 2017). *Streptococcus mutans* is a leading cariogenic bacterium as an aggressive acid/EPS producer via sugar metabolism (Bowen et al., 2018). EPSs produced by *S. mutans* are composed of polysaccharides, extracellular DNA (eDNA), lipoteichoic acids, and proteins (Pedraza et al., 2017). These molecules form scaffolds in biofilms, which act as protective barriers for bacteria (Koo et al., 2013). Polysaccharides in the matrix are mostly glucans synthesized by glucosyltransferases (Gtfs) and at least three types of Gtfs are produced by *S. mutans* as follows: GtfB (synthesizes primary insoluble glucans), GtfC (synthesizes both soluble and insoluble glucans), and GtfD (synthesizes primary soluble glucans) (Aoki et al., 1986; Hanada and Kuramitsu, 1988, 1989). During the process of biofilm formation, Gtfs secreted by *S. mutans* bind to the acquired pellicle and surfaces of non-Gtfs producers, which enables glucan production *in situ* (Schilling and Bowen, 1992; Bowen and Koo, 2011). As another crucial component of EPSs, eDNA interacts with polysaccharides and reinforces biofilms (Klein et al., 2015); moreover, it is produced by bacterial cell autolysis and membrane vesicles (MVs) in *S. mutans* (Perry et al., 2009; Liao et al., 2014). Cell autolysis in *S. mutans* is associated with holing-like proteins LrgA/B on the membrane (Ahn et al., 2010; Ahn and Rice, 2016) and MVs are the main lysis-independent mechanism through which eDNA is produced (Liao et al., 2014). Sortase A (SrtA), a transpeptidase, affects the protein profile of MVs and control eDNA production (Liao et al., 2014).

*Streptococcus mutans* is an active sugar consumer and acid producer. During the process of glycolysis, enolase (encoded by *eno*) catalyzes the production of phosphoenolpyruvate and lactate dehydrogenase (LDH, encoded by *ldh*) catalyzes the production of lactic acid. Thriving in acidic environments, *S. mutans* biofilms have the marked ability to tolerate acids due to the buffering effects of EPSs and specific agents in bacterial cells. F-type ATPase (alpha-subunit of the proton translocator is encoded by *atpD*) helps to maintain the cytoplasmic pH of *S. mutans* via H^+^ secretion under acidic conditions (Kuhnert et al., 2004). In addition, this bacterium also produces alkalis to neutralize acids via the agmatine deiminase system (the agmatine:putrescine antiporter is encoded by *aguD*) (Griswold et al., 2004, 2006).

Two-component systems (TCSs), usually comprised of histidine protein kinases and response regulator proteins, enable bacterial cells of *S. mutans* to sense environmental changes and regulate virulence factors (West and Stock, 2001). LytST TCS is responsible for the expressions of LrgA/B and autolysis of bacterial cells (Ahn et al., 2012; Florez Salamanca and Klein, 2018). VicRK TCS is associated with sucrose-dependent adhesion, biofilms formation, acid tolerance, acid production, and genetic competence of *S. mutans* due to its governor role to control the levels of GtfB/C/D, agmatine deiminase, and other virulence factors (Senadheera et al., 2005, 2009; Liu and Burne, 2009). Similarly, ComDE is involved in biofilm formation (Li et al., 2002), bacteriocin production (van der Ploeg, 2005), and acid tolerance (Gong et al., 2009; Liu and Burne, 2009). Thus, these TCSs may be the important targets for novel anti-*S. mutans* agents.

**FIGURE 1:**
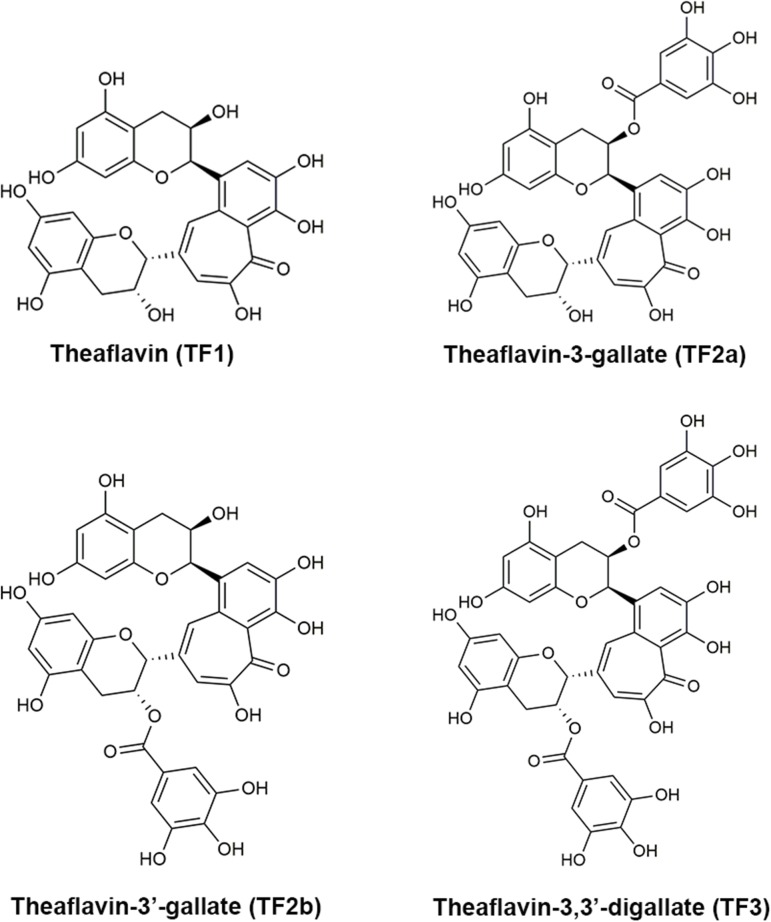
The molecular structures of theaflavin (TF1), theaflavin-3-gallate (TF2a), theaflavin-3′-gallate (TF2b), and theaflavin-3,3′-digallate (TF3).

Black tea extract has anti-caries effects both *in vitro* and *in vivo* (Linke and LeGeros, 2003; Ferrazzano et al., 2009; Barroso et al., 2018). As the most important bioactive components in black tea, theaflavins are found to inhibit the growth of *S. mutans in vitro* and decrease glucosyltransferases (Nakahara et al., 1993). However, the precise component of theaflavins that drives these effects and the mechanism underlying their anti-*S. mutans* functions have not been determined. In this study, we evaluated the anti-bacterial effects of four main theaflavins [theaflavin (TF1), theaflavin-3-gallate (TF2a), theaflavin-3′-gallate (TF2b), and theaflavin-3,3′-digallate (TF3)] (Figure 1) and found that TF3 showed the strongest inhibitory effects against *S. mutans*. Previous studies show that TF3 has strong anti-cancer (Schuck et al., 2008; Pan et al., 2018), osteoclastogenesis-repressive (Hu et al., 2017), anti-viral (Isaacs et al., 2011; de Oliveira et al., 2015), and anti-bacterial (Noor Mohammadi et al., 2018) abilities. TF3 has been observed to exhibit antibacterial effects against *Porphyromonas gingivalis*, attenuate the secretion of interleukin-8, and induce human β-defensin secretion in oral epithelial cells, suggesting that periodontal diseases may be treated by this component (Lombardo Bedran et al., 2015). However, the effects of TF3 on dental caries or *S. mutans* have not yet been studied. In this study, we found that TF3 strongly protects against the formation and cariogenic ability of *S. mutans* biofilms. We also investigated the underlying mechanisms and demonstrate the potential applications of TF3 for caries prevention.

## 2. Materials and Methods

### 2.1. Chemicals, Bacteria, and Growth Conditions

TF1 (98%), TF2a (99%), TF2b (99%), and TF3 (93%, Supplementary Figure 1) were all purchased from Biopurify Phytochemicals, Ltd. (Chengdu, China) and dissolved in ethanol to obtain solutions of the same concentration, 50 mg/mL.

*Streptococcus mutans* UA159 was amplified in Todd-Hewitt broth (THB) at 37°C in an anaerobic chamber (10% H_2_, 10% CO_2_, 80% N_2_; Bugbox, Ruskinn Technology, Bridgend, United Kingdom) for 18 h. Then, bacteria were collected by centrifugation (8,000 × *g*, 4°C, 10 min), resuspended, and diluted 1:10 in chemically-defined medium (CDM) (Socransky et al., 1985). After reaching the mid-logarithmic growth phase, the bacterial concentration was adjusted to 5 × 10^6^ colony-forming units (CFU)/mL. CDM supplemented with 1% (wt/vol) sucrose (CDMS) was used for studies of sessile bacteria.

### 2.2. Determination of the Minimum Inhibitory Concentration (MIC) and Minimum Bactericidal Concentration (MBC)

Minimum inhibitory concentration (MIC) was determined by the broth microdilution method as described previously (Wiegand et al., 2008). *S. mutans* UA159 in CDM (5 × 10^6^ CFU/mL) was treated with TF1/TF2a/TF2b/TF3, ethanol (control), or sterile water (blank) in 96-well plates. The final concentrations of four theaflavins were 0–1000 μg/mL, and the ethanol concentrations in the control group were 0–2%. The MIC was defined as the minimum concentration of an agent that inhibited the visible growth of bacteria. Bacteria in cultures were collected by centrifugation (8,000 × *g*, 4°C, 10 min), washed with phosphate-buffered saline (PBS), resuspended in CDM, and spotted onto THB agar plates (2 μL/dot). The growth of colonies was assessed on plates after 48 h of anaerobic incubation at 37°C. MBC was defined as the minimum concentration of an agent that killed all bacteria.

### 2.3. Killing Curves of TF3 Using *S. mutans*

Cultures of *S. mutans* UA159 at mid-logarithmic growth phase were adjusted to 5 × 10^6^ CFU/mL in CDM and then treated with TF3 at concentrations of 1, 2, 5, and 10× MIC. Ethanol at a concentration of 1.25% was used for the control groups, and sterile water was used for the blank groups. Concentrations of bacterial cells were measured by colony counting at 0, 1, 3, 6, 9, and 24 h.

### 2.4. Biofilm Formation and Biofilm Dispersion Assays

The capacity of TF3 to inhibit biofilm formation and disperse preformed biofilms of *S. mutans* UA159 was determined by crystal violet staining (Pierce et al., 2008; Garcia et al., 2017). For the biofilm formation assay, *S. mutans* UA159 in CDMS (5 × 10^6^ CFU/mL) was treated with TF3 at sub-MIC concentrations (1/4 or 1/2× MIC) in 96-well plates. The mixture was incubated under anaerobic conditions at 37°C for 24 h. A biofilm dispersion assay was performed by adding TF3 at various concentrations (1, 2, 5, or 10× MIC) for 16 h to biofilms preformed for 8 h in 96-well plates (Garcia et al., 2017). Ethanol at the corresponding concentrations (0.0625% for biofilm formation assay and 1.25% for biofilm dispersion assay) was used for the control groups, and sterile water was used for the blank groups. After treatment, non-adherent bacterial cells were removed by gently washing the wells with PBS three times. Then, the wells were treated with methanol for 15 min and subsequently stained with a crystal violet solution (1% crystal violet; Sigma-Aldrich, St. Louis, MO, United States) for 5 min. PBS was used to wash the wells until the blank wells appeared colorless, and 95% ethanol was then added for 30 min. Absorbance was measured using a microplate reader (Molecular Devices, Sunnyvale, CA, United States) at 595 nm.

### 2.5. Quantification of Dextran Formation

Cascade Blue-conjugated dextran dye (500 nmol/L; Thermo Fisher Scientific, Waltham, MA, United States; excitation, 400 nm; emission, 420 nm) was added to CDMS prior to inoculation (Garcia et al., 2017). *S. mutans* UA159 (5 × 10^6^ CFU/mL) in 96-well plates was treated with TF3 (1/4 or 1/2× MIC); 0.0625% ethanol was used for the control groups, and sterile water was used for the blank groups. The plates were incubated under anaerobic conditions at 37°C for 24 h. Then, the fluorescence of dextran was measured by a microplate reader from the bottom of the plates without removing planktonic bacteria.

**Table 1:** Primers for PCR semiquantification of eDNA expression.

Primers	Sequences (5′–3′)
16S rRNA	8F: AGAGTTTGATCCTGGCTCAG 1510R: GGCTACCTTGTTACGA


### 2.6. Semi-Quantification of eDNA Expression in the Biofilm Matrix

The eDNA level in the matrix of biofilms was detected by PCR with specific primers for the 16S rRNA gene (Table 1) (Jung et al., 2017). *S. mutans* UA159 (5 × 10^6^ CFU/mL in CDMS) was grown in tubes (200 μL; Axygen, Tewksbury, MA, United States) with TF3 (1/4 or 1/2× MIC) for 6 or 24 h; 0.0625% ethanol was used for the control groups, and sterile water was used for the blank groups. Then, biofilms that formed on the tube walls were dispersed by vortexing. The cell-free supernatant was isolated by centrifugation (14,000 × *g*, 4°C, 10 min) and passed through a 0.22-μm filter. One microliter of the supernatant was used as a template. The 25-μL reaction system contained 1× Taq PCR Master mix (Thermo Fisher Scientific) and 10 μmol/L forward primers and reverse primers. DNA bands were obtained by 1% agarose gel electrophoresis of the PCR products after 30 cycles. Semi-quantification was performed using ImageLab 3.0.

### 2.7. Acid Production and Acid Tolerance Assays

The effects of TF3 on the acid-producing ability of *S. mutans* UA159 biofilms were determined according the methods described by Pandit et al. (2014) and Dang et al. (2016). Biofilms of *S. mutans* UA159 preformed for 24 h in 24-well plates were washed three times with PBS and treated with PBS containing TF3 (1× MIC) at 37°C for 24 h; 0.125% ethanol was used for the control groups, and sterile water was used for the blank groups. Then, the biofilms were washed three times and treated with a salt solution (50 mmol/L KCl and 1 mmol/L MgCl_2_, pH 7.0). The pH of the supernatant was adjusted to 7.2 using 0.2 mol/L KOH, and 1% (wt/vol) glucose was added. Decreases in the pH of the supernatants were assessed using a pH meter (Sartorius PB-10, Göttingen, Germany) for 0–120 min, and the final pH was measured after 360 min. For the acid tolerance assay, biofilms of *S. mutans* UA159 were preformed in 96-well plates (colony counting) or on 10 mm × 10 mm glass coverslips in 24-well plates [confocal laser scanning microscopy (CLSM)] for 24 h. After washing with PBS three times, biofilms were treated with TF3 and acid separately or in combination for 24 h. Bactericidal effects were estimated by colony counting and CLSM.

### 2.8. Visualization of Biofilms by Field Emission-Scanning Electron Microscopy (FE-SEM)

*Streptococcus mutans* UA159 (5 × 10^6^ CFU/mL) in CDMS was grown with TF3 (1/4× MIC) for 24 h on 10 mm × 10 mm glass coverslips in 24-well plates. Biofilms in control groups were grown with 0.031% ethanol. After washing with PBS three times, the biofilms were fixed in 2.5% glutaraldehyde in PBS overnight at 4°C, washed with PBS, and dehydrated using a graded ethanol series (25, 50, 75, 75, 90, 90, 100, and 100%), each for 15 min. Then, the biofilms were fixed with hexamethyldisilazane (Sigma-Aldrich) (three times, each for 5 min) and dried in a desiccator overnight. The biofilms were analyzed using a Nova NanoSEM 450 field emission-scanning electron microscope (FEI, Ltd., Hillsboro, OR, United States) under an acceleration voltage of 500 V.

### 2.9. Visualization of Biofilms by Confocal Laser Scanning Microscopy

For the biofilm formation assay, 500 nmol/L Cascade Blue-conjugated dextran dye was added to CDMS prior to inoculation. SYTO 9 (1 μmol/L; excitation, 480 nm; emission, 500 nm) and propidium iodide (PI; 0.67 μmol/L; excitation, 490 nm; emission, 635 nm) were added to stain bacteria and eDNA respectively, after incubation (Garcia et al., 2017). All images were acquired using a Nikon confocal microscope with a 10 or 60× objective lens.

### 2.10. RNA Extraction

Bacteria in biofilms were collected by pipetting, which was followed by centrifugation (8,000 × *g*, 4°C, 5 min) and washing with ice-cold PBS. RNA was stabilized using the RNAprotect Bacteria Reagent (Qiagen, Valencia, CA, United States) before extraction. The bacteria were then treated with tris-HCl (pH 8.0) containing 20 mg/mL lysozyme and 20 mg/mL proteinase K at 37°C for 60 min (Xu et al., 2011). Spheroplasts of *S. mutans* UA159 were collected by centrifugation (8,000 × *g*, 4°C, 5 min) and resuspended in TRIzol reagent (Invitrogen, Carlsbad, CA, United States). The cells were disrupted by aggressive pipetting, and RNA was extracted using chloroform. Reverse transcription was performed using the PrimeScript^TM^ RT Reagent Kit with gDNA eraser to ensure no contamination (Takara, Tokyo, Japan).

### 2.11. Quantitative Real-Time PCR (RT-PCR)

The expression levels of *gtfB, gtfC, gtfD, vicK, vikR, lrgA, lrgB, lytS, lytT, srtA, ldh, eno, comD, comE, atpD*, and *aguD* mRNA were quantified by RT-PCR. Primers are listed in Table 2. The expression of 16S rRNA was used as an internal control. Amplification was performed on the ViiA^TM^ 7 Real-Time PCR System (Applied Biosystems, Foster City, CA, United States). The 25-μL reaction system contained 1× SYBR green PCR Master Mix (Takara), 1 μL of template cDNA, and 10 μmol/L forward and reverse primers. The two-step PCR (*gtfB, gtfC, gtfD, vicK, vikR, lytT, srtA, ldh, eno, comD, comE, atpD*, and *aguD*) included 95°C for 15 s and 60°C for 1 min (40 cycles) and three-step PCR (*lrgA, lrgB*, and *lytS*) included 95°C for 15 s, 53°C for 30 s, and 72°C for 30 s (40 cycles). The steps of melting curves were 95°C for 30 s, and an increase from 55 to 95°C over 10 s. Cycle threshold (CT) values were determined using ViiA 7 RUO, and data were analyzed by the 2^-ΔΔCT^ method.

**Table 2:** Primers for RT-PCR.

Primers	Sequences (5′–3′)
16S rRNA (Xu et al., 2012)	F: AGCGTTGTCCGGATTTATTG R: CTACGCATTTCACCGCTACA
*gtfB* (Xu et al., 2012)	F: CACTATCGGCGGTTACGAAT R: CAATTTGGAGCAAGTCAGCA
*gtfC* (Xu et al., 2012)	F: GATGCTGCAAACTTCGAACA R: TATTGACGCTGCGTTTCTTG
*gtfD* (Xu et al., 2012)	F: TTGACGGTGTTCGTGTTGAT R: TTGACGGTGTTCGTGTTGAT
*lrgA* (Florez Salamanca and Klein, 2018)	F: GTCTATCTATGCTGCTATT R: AAGGACATACATGAGAAC
*lrgB* (Florez Salamanca and Klein, 2018)	F: GTAAAGGCTTCTTTCTCT R: TAACATCAGTTCCCAATC
*lytS* (Florez Salamanca and Klein, 2018)	F: TTCAGAGACTTGGTATTATC R: TTGGAAATGATGACGAAA
*lytT* (Florez Salamanca and Klein, 2018)	F: TGGCAAGACAAGAGTTAA R: GCTAATATCTTCAGCTTCAA
*srtA* (Zhou et al., 2018)	F: GAAGCTTCCTGTAATTGGCG R: TTCATCGTTCCAGCACCATA
*ldh* (Xu et al., 2011)	F: GGCGACGCTCTTGATCTTAG R: GGTTAGCAGCAACGAGGAAG
*eno* (Xu et al., 2011)	F: CAGCGTCTTCAGTTCCATCA R: TCACTCAGATGCTCCAATCG
*atpD* (Xu et al., 2011)	F: TGTTGATGGTCTGGGTGAAA R: TTTGACGGTCTCCGATAACC
*aguD* (Xu et al., 2011)	F: TGGTGCTGCTCTTGCTAATG R: TAAAAGGACGCGGTGTATCC
*comD* (Chakraborty and Burne, 2017)	F: TATGGTCTCTGCCTGTTGC R: TGCTACTGCCCATTACAATTCC
*comE* (Stipp et al., 2008)	F: ATTGACGCTATCCCTGAAAAG R: TGAAAAGTGAGGGGCATAAA
*vicK* (Senadheera et al., 2005)	F: CACTTTACGCATTCGTTTTGCC R: CGTTCTTCTTTTTCCTGTTCGGTC
*vicR* (Senadheera et al., 2005)	F: CGCAGTGGCTGAGGAAAATG R: ACCTGTGTGTGTCGCTAAGTGATG


### 2.12. Statistical Analysis

All experiments were performed in triplicate and independently reproduced at least three times. Data were shown in Supplementary Tables 1–11 and analyzed using SPSS (version 22.0 for Windows). One-way ANOVA and two-way ANOVA were performed. *P* < 0.05 was considered statistically significant.

## 3. Results

### 3.1. The Antibacterial Effects of TF3 on Planktonic *S. mutans* UA159

#### 3.1.1. TF3 Exerts the Strongest Antibacterial Effects Among Four Theaflavins

To clarify which component of theaflavins drives the anti-bacterial effects against *S. mutans*, four main theaflavins (TF1/TF2a/TF2b/TF3) were investigated and MICs were determined (Supplementary Figure 2). TF1 inhibited the growth of *S. mutans in vitro* in CDM with an MIC of 500 μg/mL, which was higher than that of TF2a and TF2b (both 125 μg/mL). TF3 had the lowest MIC of 62.5 μg/mL among the four components and the MBC was 125 μg/mL (Supplementary Figure 3).

#### 3.1.2. Killing Curves of TF3 Using *S. mutans*

**FIGURE 2:**
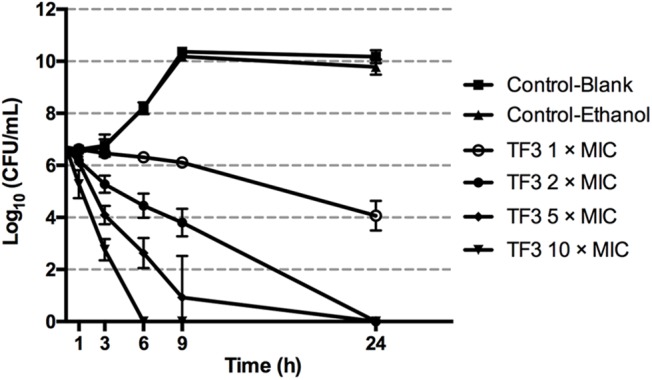
Killing curves of TF3 on *Streptococcus mutans* UA159 in CDM. The initial bacterial concentration was 5 × 10^6^ CFU/mL. Concentrations of bacterial cells were determined by colony counting after treatment with TF3 at 1, 2, 5, and 10× MIC for 1, 3, 6, 9, and 24 h. The results are given as means and standard deviations from three independent experiments.

Figure 2 shows the killing curves of TF3 using *S. mutans* UA159. With 62.5 μg/mL TF3 treatment, an overall 2.63-log_10_ reduction was noted at 24 h, corresponding to 99.65% killing of live cells. The bactericidal effect was also dose-dependent. A 2.24-log_10_ reduction (99.22% killing) and 2.90-log_10_ reduction (99.81% killing) was achieved after 6 and 9 h of treatment, respectively, with 125 μg/mL TF3. With 312.5 μg/mL TF3 treatment, a 2.60-log_10_ reduction (99.70% killing) of bacterial cells was observed after 3 h and almost all cells were killed by 6 h (99.98% killing). At the concentration of 625 μg/mL, TF3 killed 93.41 and 99.98% of *S. mutans* after 1 and 3 h of treatment, respectively.

#### 3.1.3. The Bactericidal Effects of TF3 at Concentrations Below the MIC

**FIGURE 3:**
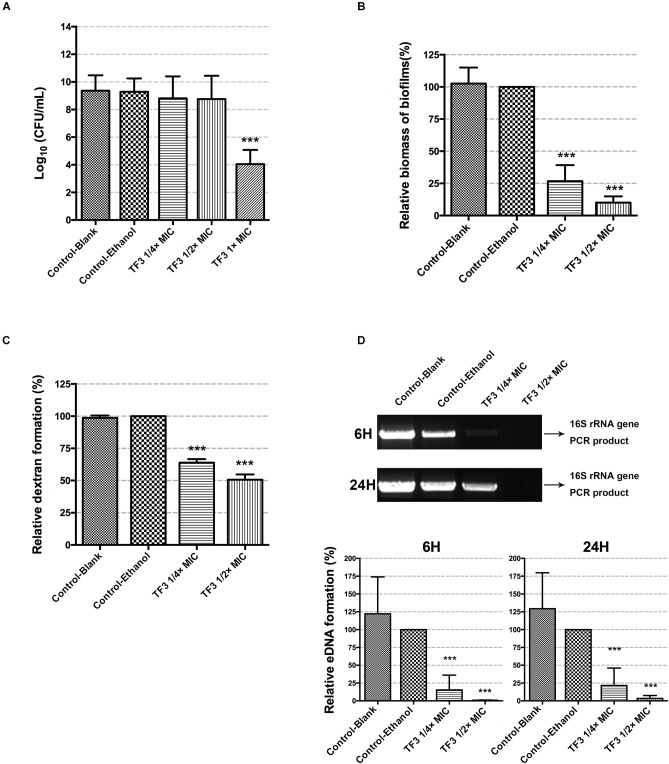
The bactericidal effects of sub-MIC TF3 on *S. mutans* UA159 were determined by colony counting **(A)**. The effects of TF3 on *S. mutans* UA159 biofilm formation were detected by crystal violet staining and OD595 measurements. Data shown is relative biomass of biofilms compared with the control-ethanol group **(B)**. Dextran formation of *S. mutans* UA159 was detected by measuring the fluorescence excited by a Cascade Blue-conjugated dextran dye. Data shown is relative dextran formation compared with the control-ethanol group **(C)**. The eDNA content in biofilms of *S. mutans* UA159 was semi-quantified by PCR using primers for the 16S rRNA gene. The PCR products were imaged using an ultraviolet camera after electrophoresis on 1% agarose gels and quantified using Image Lab. The full scans of the original gels are shown in Supplementary Figure 5. Data shown is relative eDNA formation compared with the control-ethanol group **(D)**. The results are shown as the mean and standard deviation of three independent experiments. ^∗∗∗^*P* < 0.001, when compared with the control-ethanol group using one-way ANOVA.

To investigate the bacterial killing effect of TF3 against *S. mutans* at concentrations below the MIC, bacteria in CDM (5 × 10^6^ CFU/mL) were treated with 1/4 or 1/2× MIC TF3 for 24 h. Concentrations of bacterial cells in CDM at the endpoint were determined by colony counting. The results shown in Figure 3A indicated that the concentrations of living bacterial cells in groups treated with 1/4 or 1/2× MIC TF3 were not significantly different than that in control (0.0625% ethanol).

### 3.2. Biofilm Formation of *S. mutans* UA159 Is Significantly Inhibited by TF3

To determine whether TF3 inhibited the biofilm formation of *S. mutans* without bactericidal effects, biofilms were grown with TF3 at concentrations below the MIC (1/4 or 1/2× MIC). As shown in Figure 3B, the biomass of biofilms in the 15.6 μg/mL (1/4× MIC) TF3 group was less than 50% and in the 31.25 μg/mL (1/2× MIC) TF3 group was less than 25% of that in the ethanol control group.

**FIGURE 4:**
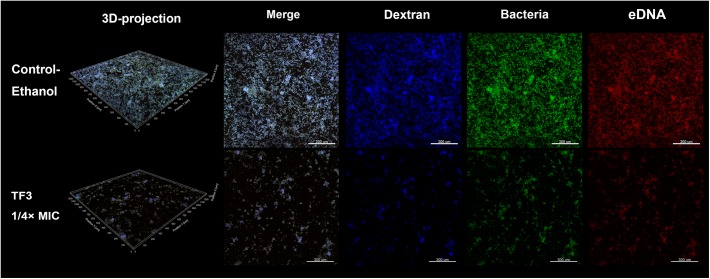
Biofilms of *S. mutans* UA159 in the control-ethanol (0.031%) group and TF3 (1/4× MIC) group were visualized by CLSM. Biofilms assessed by CLSM were grown with a Cascade Blue-conjugated dextran dye, and bacteria and eDNA were labeled with SYTO 9 and PI, respectively. The biofilms were imaged with a 10× objective lens.

**FIGURE 5:**
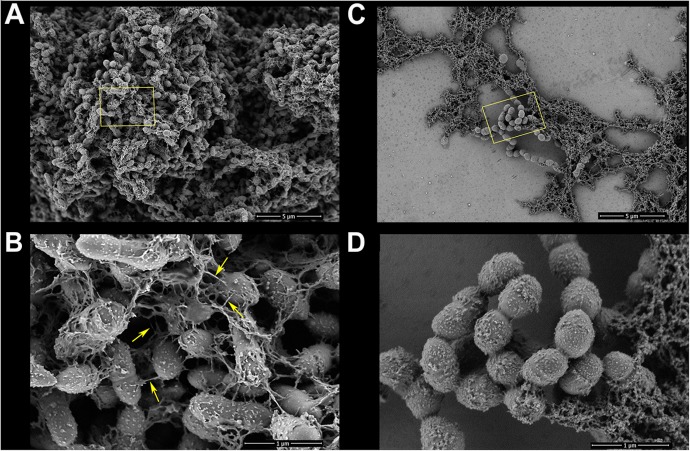
Biofilms of *S. mutans* UA159 in the control-ethanol (0.031%) group and TF3 (1/4×MIC) group were visualized by FE-SEM. Panels **(A)** and **(B)** show biofilms in the control group at magnifications of 5,000 and 30,000×, respectively. Bacterial cells in the control group exhibited substantial surrounding EPS matrixes (yellow arrows show nanofibers connecting bacterial cells). Panels **(C)** and **(D)** show biofilms grown with TF3 (1/4×MIC) at magnifications of 5,000 and 30,000×, respectively.

The two main component of EPS, dextran and eDNA, were also quantified and visualized to explore the mechanisms underlying these effects. As shown in Figure 3C,D, dextran and eDNA levels were significantly lower in groups grown with TF3 (1/4 or 1/2× MIC) than in the control group (*P* < 0.001). With biofilm maturation, eDNA accumulated. However, the eDNA produced in the TF3 groups was still less than that produced in the control group at 24 h. The same results were obtained by CLSM (Figure 4) and field emission-scanning electron microscopy (FE-SEM) (Figure 5). The blue fluorescence of dextran and red fluorescence of eDNA were lower for biofilms grown with 15.6 μg/mL TF3 than with 0.031% ethanol (Figure 4). Biofilms grown with 15.6 μg/mL TF3 were also less dense and had fewer connections between bacterial cells (Figure 5). Further, RT-PCR results indicated that TF3 (1/4 or 1/2× MIC) could significantly downregulate the expression levels of *gtfB, gtfC, gtfD, lrgA, lrgB*, and *srtA* (Figure 9; *P* < 0.001).

### 3.3. TF3 Is Effective Against Established *S. mutans* UA159 Biofilms

**FIGURE 6:**
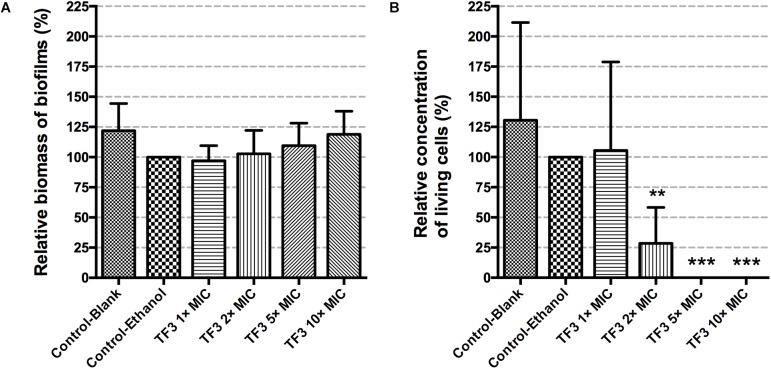
The biofilm dispersion effects of TF3 on established *S. mutans* UA159 biofilms were detected by crystal violet staining and OD595 measurements. Data shown is relative biomass of biofilms compared with the control-ethanol group **(A)**. The bactericidal efficiency of TF3 at different concentrations against established *S. mutans* UA159 biofilms was determined by colony counting. Data shown is relative concentration of living cells compared with the control-ethanol group **(B)**. The results are shown as the mean and standard deviation of three independent experiments. ^∗∗^*P* < 0.01 and ^∗∗∗^*P* < 0.001, when compared with the control-ethanol group using one-way ANOVA.

**FIGURE 7:**
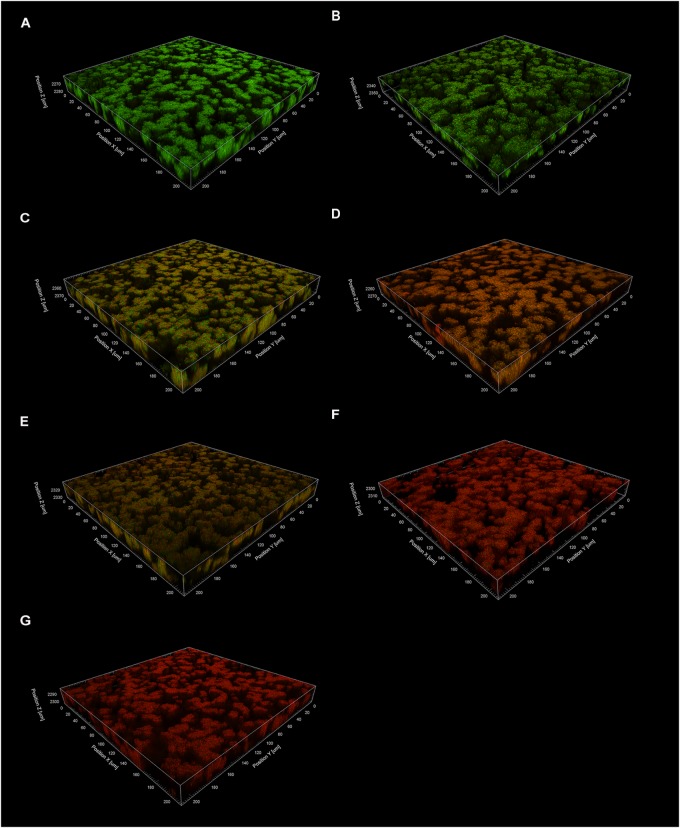
The effects of TF3 on established *S. mutans* UA159 biofilms were visualized by CLSM. Biofilms were treated with ethanol (control) **(A)**, acid (pH = 1.5) **(B)**, TF3 (1× MIC) **(C)**, TF3 (1× MIC) and acid (pH = 1.5) **(D)**, TF3 (2× MIC) **(E)**, TF3 (5× MIC) **(F)** or TF3 (10× MIC) **(G)**, stained with SYTO 9 and PI, and imaged with a 60× objective lens.

Mature biofilms of *S. mutans* are usually too firm to be eliminated and have stronger ability to tolerate anti-bacterial agents due to abundant EPS. One agent has been reported to disperse preformed biofilms of *S. mutans* and is expected to have potential for the prevention of caries (Garcia et al., 2017). The ability of TF3 to disperse biofilms generated by *S. mutans* UA159 was studied and found no effect (*P* > 0.05; Figure 6A). Then, the bactericidal effects of TF3 on established biofilms were evaluated. Wells containing biofilms of *S. mutans* formed for 24 h in 96-well plates were treated with CDM containing 1, 2, 5, or 10× MIC TF3 at 37°C for 24 h. As determined by colony counting assays and CLSM, TF3 could kill almost 100% of bacteria in the biofilms at 5 and 10× MIC and more than 50% of the bacteria at 2× MIC after 24 h, but no significant bactericidal effect was observed at 1× MIC (Figure 6B). These results were also visualized by CLSM (Figure 7).

#### 3.3.1. TF3 Negatively Affects the Acid Production and Acid Tolerance of *S. mutans* UA159 Biofilms

**FIGURE 8:**
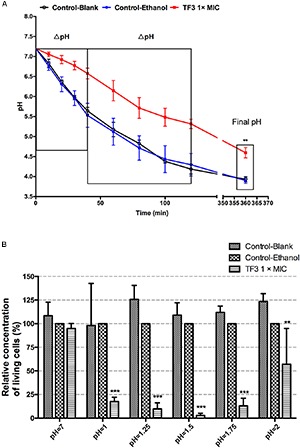
Acid production of *S. mutans* UA159 biofilms were detected in salt solutions with 1% glucose. Biofilms were pre-treated with TF3 (1× MIC), 0.125% ethanol or sterile water for 24 h. Decreases in pH of the supernatants were assessed using a pH meter. The ΔpH was calculated at 0–40 and 40–120 min and the final pH (360 min) was recorded **(A)**. The bactericidal efficiency of TF3 at 1× MIC against established *S. mutans* UA159 biofilms under different pHs was determined by colony counting. Data shown is relative concentration of living cells compared with the control-ethanol group **(B)**. The results are shown as the mean and standard deviation of three independent experiments. ^∗∗^*P* < 0.01 and ^∗∗∗^*P* < 0.001, when compared with the control-ethanol group using using two-way ANOVA.

**FIGURE 9:**
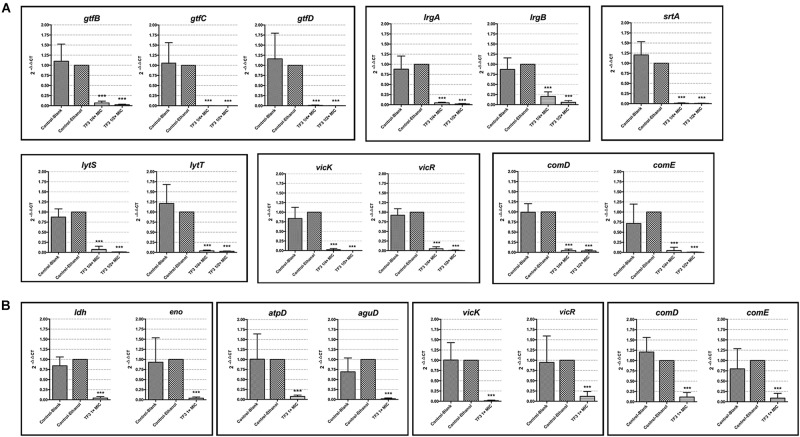
The mRNA expression levels of *gtfB, gtfC, gtfD, lrgA, lrgB, srtA, lytS, lytT, vicK, vikR, comD*, and *comE* in planktonic bacterial cells of *S. mutans* UA159 grown with 1/4 or 1/2× MIC TF3 for 24 h were quantified by RT-PCR **(A)**. The mRNA expression levels of *ldh, eno, atpD, aguD, vicK, vikR, comD*, and *comE* in bacterial cells in preformed biofilms after 24 h-treatment with 1× MIC TF3 were quantified by RT-PCR **(B)**. The expression levels of all target genes were normalized using the 16S rRNA as internal control. The results are shown as the mean and standard deviation of three independent experiments. ^∗∗∗^*P* < 0.001, when compared with the control-ethanol group using using one-way ANOVA.

Biofilm is the main lifestyle of *S. mutans*. The ability of *S. mutans* biofilms to produce and tolerate acid is the main cause of enamel demineralization. The effects of TF3 on the acid production and acid tolerance of *S. mutans* UA159 biofilms are summarized in Figure 8. The concentration (1× MIC) at which TF3 showed no significant bactericidal effect was used. The acid production rate was estimated as the decrease in the pH value (ΔpH) of the supernatant during a specific period. At 0–40 and 40–120 min, the ΔpH values for the biofilms treated with TF3 (1× MIC) for 24 h were significantly lower than those of the control biofilms (*P* < 0.05). The final pH values (at 360 min) of the supernatants in the wells treated with TF3 were also significantly higher than those of the control supernatants (*P* < 0.01; Figure 8A), which might be caused by the diminished acid tolerance of bactericidal cells. To verify this, biofilms were treated with TF3 and acid separately or in combination and the bactericidal efficiency was calculated by colony counting. Biofilms treated with both TF3 (1× MIC) and acid (pH = 1–2) had lower relative living cells than those treated with TF3 or acid alone (*P* < 0.01; Figure 8B). The results were also confirmed by CLSM. As shown in Figure 7, the combined treatment of biofilms with both TF3 (1× MIC) and acid (pH = 1.5) resulted in stronger red fluorescence than that with separate treatments of TF3 or acid. RT-PCR results confirmed that bacterial cells in *S. mutans* biofilms treated with TF3 (1× MIC) had much lower mRNA expression levels of *ldh, eno, atpD*, and *aguD* than those in control-treated biofilms (*P* < 0.001; Figure 9).

### 3.4. TF3 Downregulates the Expression Levels of Genes in the Two-Component Systems

Two-component systems are considered mechanisms through which *S. mutans* senses extracellular factors and regulates virulence factors. To better understand the regulatory effect of TF3 on *S. mutans*, the expression levels of genes involved in the TCSs were revealed by RT-PCR (Figure 9). The results showed that planktonic bacterial cells grown with 1/4 or 1/2× MIC TF3 had lower expression levels of *vicK, vicR, lytS, lytT, comD*, and *comE* (*P* < 0.001). TF3 also downregulated the expression levels of *vicK, vicR, comD*, and *comE* (*P* < 0.001) in bacterial cells in preformed biofilms after 24 h-treatment.

## 4. Discussion

The present study investigated the anti-bacterial effects of four components of theaflavins on *S. mutans* and found that TF3 had the lowest MIC, indicating that it has the strongest anti-bacterial effects against *S. mutans*. The results are consistent with a previous study of the four same theaflavins using *Bacillus cereus* (Friedman et al., 2006). The killing effect of TF3 against *S. mutans* was found to be dose-dependent; TF3 at concentrations equal to or higher than 2× MIC killed all bacterial cells of *S. mutans* after 24 h. However, after treatment for 1 h, a greater than 90% killing effect was only observed at 10× MIC (Figure 2). Similarly, TF3 at 10× MIC killed all bacterial cells in preformed biofilms of *S. mutans* after 24 h, but no significant bactericidal effect was observed for TF3 toward preformed biofilms after treating them for 5 min (Supplementary Figure 4). The results suggest that the killing effect of TF3 against *S. mutans* relies on high concentrations and sufficient time.

However, TF3 is found to be an efficient anti-biofilm agent against *S. mutans* at sub-bactericidal concentrations. Dental caries is not caused by one or several specific pathogens but is associated with the dysbiosis of the local microbiome (Ling et al., 2010; Wang et al., 2017). Many factors cause this dysbiosis, among which *S. mutans* plays critical roles in dental plaque formation and the transformation of the normal microbial ecosystem into a cariogenic state (Loesche, 1986; Gross et al., 2012). Therefore, an ideal anti-caries agent suppresses the virulence factors of *S. mutans* at concentrations that are too low to cause broad-spectrum bactericidal effects. In this study, we found that TF3 inhibits *S. mutans* UA159 biofilm formation, without affecting bacterial growth. Similarly, TF3 diminished the ability of *S. mutans* biofilms to produce and tolerate acid, without significant bactericidal effects. These findings suggested that it is a potentially effective anti-caries agent.

The inhibitory effect of TF3 on the biofilm formation of *S. mutans* was attributed to the decreased dextran and eDNA (Figure 3). Dextran is a glucan formed by *S. mutans* and is crucial for the establishment of cariogenic biofilms (Schilling and Bowen, 1992). TF3 efficiently suppressed the expression of genes (*gtfB, gtfC*, and *gtfD*) encoding Gtfs (Figure 9), suggesting that it can decrease the formation of both soluble and insoluble glucans, which include dextran. The results are consistent with a previous study, which found that theaflavins inhibit the Gtfs of *S. mutans* (Nakahara et al., 1993). eDNA has a vital role in biofilm formation (Whitchurch et al., 2002; Das et al., 2010). To our knowledge, few other agents have been demonstrated to decrease eDNA production by *S. mutans*. Two routes for eDNA production in *S. mutans* have been reported, namely autolysis (Perry et al., 2009) and secretion via MVs (Liao et al., 2014). The pathway for bacterial cell autolysis has been clarified in *S. mutans* (LytST TCS regulates LrgA/B), but the genetic network for eDNA secretion by MVs is unclear. Previously, one study has shown that mutations in *srtA* change components of MVs and reduce eDNA (Liao et al., 2014). Our data suggested that TF3 downregulates eDNA production by *S. mutans* by suppressing bacterial cell autolysis and affecting secretion systems.

Once biofilms are formed, it is very different to eliminate them. Biofilm dispersion is a mode of biofilm detachment, which causes individual cells to separate from the biofilms (Kim and Lee, 2016). In present study, we found no disperse effect of TF3 against established biofilms of *S. mutans* (Figure 6A). Therefore, the reduction of amount of living bacterial cells in TF3-treated biofilms (Figure 6B) can be attributed to the bactericidal effects of TF3, rather than dispersion of bacterial cells. Although the bactericidal effect of TF3 at 1× MIC on preformed biofilms was not obvious, the acid production and acid tolerance of biofilms were inhibited. In addition to the scaffolding matrix of biofilms, the acid production and acid tolerance of *S. mutans* bacterial cells are the key targets of anti-caries agents (Pandit et al., 2014). The diminished effect of TF3 on acid production of *S. mutans* might explain the previous study which found that oolong tea extract reduced the rate of acid production in *S. mutans* (Matsumoto et al., 1999). RT-PCR showed that *S. mutans* treated with 1× MIC TF3 expressed lower levels of *ldh, eno, atpD*, and *aguD* than control-treated *S. mutans.* It must be noted that *ldh* encodes LDH, *eno* encodes enolase, *atpD* is responsible for the expression of the alpha-subunit of the proton translocator, and *aguD* encodes the agmatine:putrescine antiporter of the agmatine deiminase system (Xu et al., 2011). Thus, by suppressing these specific virulence factors, TF3 inactivated acid production and reduced acid tolerance in biofilms.

As discussed previously herein, TF3 suppressed biofilm formation, acid production, and acid tolerance in *S. mutans* by targeting specific virulence factors. However, the regulatory network behind these effects still needs to be revealed. TCSs receive extracellular signals and regulate intracellular factors. They play major roles in quorum sensing systems and environmental stress responses. In *S. mutans*, two TCSs are clearly presented, namely VicRK and ComDE. VicRK TCS, comprised of VicK histidine kinase and VicR response regulator, regulates the expression of *gtfBCD, gbpB*, and *ftf*, which consequently affects biofilm formation (Senadheera et al., 2005). In addition, VicRK TCS is also related to the stress response of *S. mutans*. A VicK-null mutant exhibits decreased lactate production and impaired acid tolerance response, with the downregulation of genes encoding the ATPase subunit and agmatine deiminase when exposed to low pH (Liu and Burne, 2009; Senadheera et al., 2009). However, the survival rate of the VicK-deficient strain was found to be enhanced at low pH due to improved resistance to autolysis (Senadheera et al., 2009). VicK was later found to be responsible for calcium ion sensing and subsequent AtlA-mediated autolysis (Jung et al., 2017). The ComDE TCS, comprised of ComD histidine kinase and ComE response regulator, is a vital component of quorum sensing mechanisms, which senses competence-stimulating peptides (CSPs) (Havarstein et al., 1996). The functions of ComDE go beyond genetic competence and bacteriocin production (van der Ploeg, 2005). Acid tolerance is regulated by ComDE via agmatine deiminase gene expression (Liu and Burne, 2009). Moreover, the deletion of *comD* or *comE* results in reduced biofilm biomass in *S. mutans*, indicating that the ComDE TCS is essential for biofilm formation (Li et al., 2002). Therefore, TCSs may have potential to be effective targets of anti-*S. mutans* agents. Of note, some natural agents have been found to suppress TCSs in *S. mutans* (Zhang et al., 2015; Li et al., 2018), but no similar studies have been reported on black tea extracts or theaflavins. In the present study, based on RT-PCR results, we speculate that TF3 suppresses at least three TCSs in *S. mutans* (LytST, VicRK, and ComDE), which might explain its inhibitory effect on biofilm formation, acid tolerance and acid production.

**FIGURE 10:**
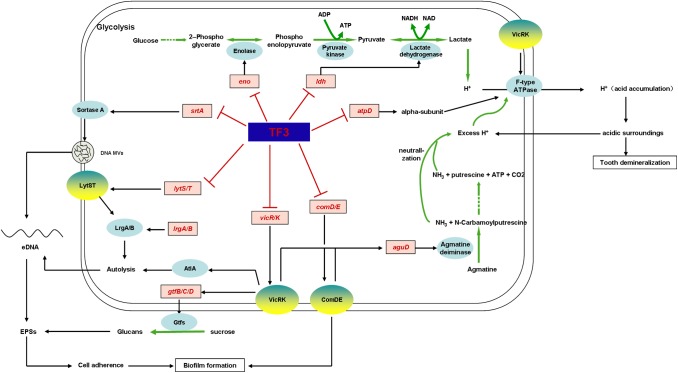
Mechanistic effects of TF3 on *S. mutans* was revealed by RT-PCR and summarized. TF3 down-regulated the expression levels of genes involved in biofilm formation, acid production, and acid tolerance (genes in red).

Thus, we were able to summarize the mechanism underlying the anti-*S. mutans* effects of TF3 (Figure 10). TF3 at sub-bactericidal concentrations downregulates the expression of *lytS, lysT, vicR, vicK, comD*, and *comE*, which consequently disturbs the functions of LytST, VicRK, and ComDE TCSs. When cell density increases, bacterial cells fail to communicate with each other due to the suppressed ComDE TCS. Restrained LrgA/B and AtlA will be caused by the suppression of LytST and VicK, respectively, which leads to resistance to cell autolysis. Moreover, eDNA MVs are affected by the inhibition of SrtA. As a consequence, the amount of eDNA decreases. The suppression of VicRK also inhibits glucan synthesis by GtfB/C/D. Without efficient cooperation, cells of *S. mutans* produce few EPSs (eDNA and glucans) and are unable to form mature biofilms.

Moreover, TF3 at sub-bactericidal concentrations was found to diminish the acid production and acid tolerance of *S. mutans* biofilms via the downregulation of enzymes involved in glycolysis (lactic dehydrogenase encoded by *ldh*, enolase encoded by *eno*) and the maintenance of cytoplasmic pH (F-type ATPase and the agmatine deiminase system). The inhibition of the agmatine deiminase system might be achieved by the suppression of VicRK and ComDE, whereas the downregulation of F-type ATPase might be associated with VicK.

Our findings provide a scientific basis for the application of TF3 as an anti-caries agent. However, this study only explores the inhibitory effects of TF3 on monospecies biofilm, namely that of *S. mutans, in vitro*. The *in vivo* microbiome is far more complicated, and the surroundings are different in the oral cavity. Further studies are thus needed to explore the inhibitory effects of TF3 on the multispecies biofilm *in vitro* and *in vivo*.

## 5. Conclusion

In summary, TF3 is a bactericidal agent against *S. mutans* which need high concentrations and sufficient time. However, the role as an anti-biofilm agent is more important of TF3. TF3 at sub-inhibitory concentrations suppresses *S. mutans* biofilm formation by reducing glucan and eDNA production. These effects could be attributed to the decreased generation of Gtfs, inhibited cell autolysis, and alterations in MVs components. In addition, TF3 at sub-bactericidal concentrations suppressed the acid production and acid tolerance of *S. mutans* biofilms via the downregulation of enzymes involved in glycolysis and the maintenance of cytoplasmic pH. The mechanisms were further revealed to be associated with the downregulation of LytST, VicRK, and ComDE two component systems.

## Data Availability

The datasets generated for this study are available on request to the corresponding author.

## Author Contributions

SW, YuW, YiW, and ZD carried out the experiments. SW, YuW, and WW analyzed the data and wrote the original draft of the manuscript. YiW and ST provided the technical support. SD and HW conceived the project and designed the experiments. ZL, SD, and HW revised and finalized the manuscript.

## Conflict of Interest Statement

The authors declare that the research was conducted in the absence of any commercial or financial relationships that could be construed as a potential conflict of interest.
