# Supplementary material for: Theaflavin-3,3′-Digallate Suppresses Biofilm Formation, Acid Production, and Acid Tolerance in Streptococcus mutans by Targeting Virulence Factors
Source: Front Microbiol. 2019 Jul 26;10:1705. doi: 10.3389/fmicb.2019.01705 (PMC6676744; doi:10.3389/fmicb.2019.01705)
Supplement: Supplementary file 1 [file Table_1.DOCX]

Supplementary Material


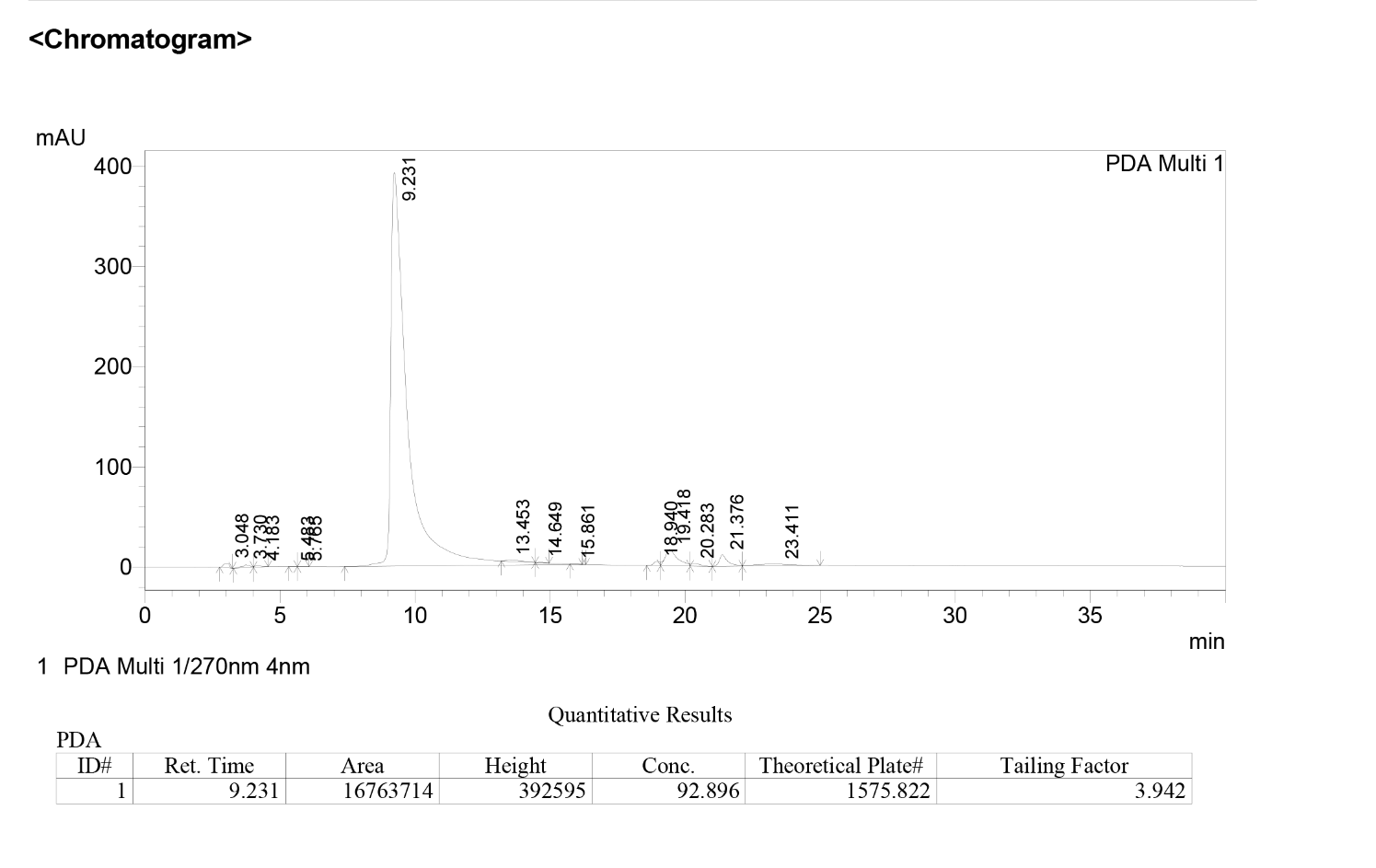


**Supplementary Figure 1.** High performance liquid chromatography (HPLC) analysis of TF3 powder. Column: Ultimate@ AQ-C18(4.6mm*250mm, 5um); column temperature: 30°C; detection mode: UV 270nm; flow rate: 1.0 mL/min; sample dissolution: methanol; mobile phase: acetonitrile (A) and 0.1% formic acid in water (B); gradient elution: 0–5min (A : B = 30 : 70–60 : 40), 5–15min (A : B = 60 : 40–85 : 15), 15–40min (A : B=30 : 70).


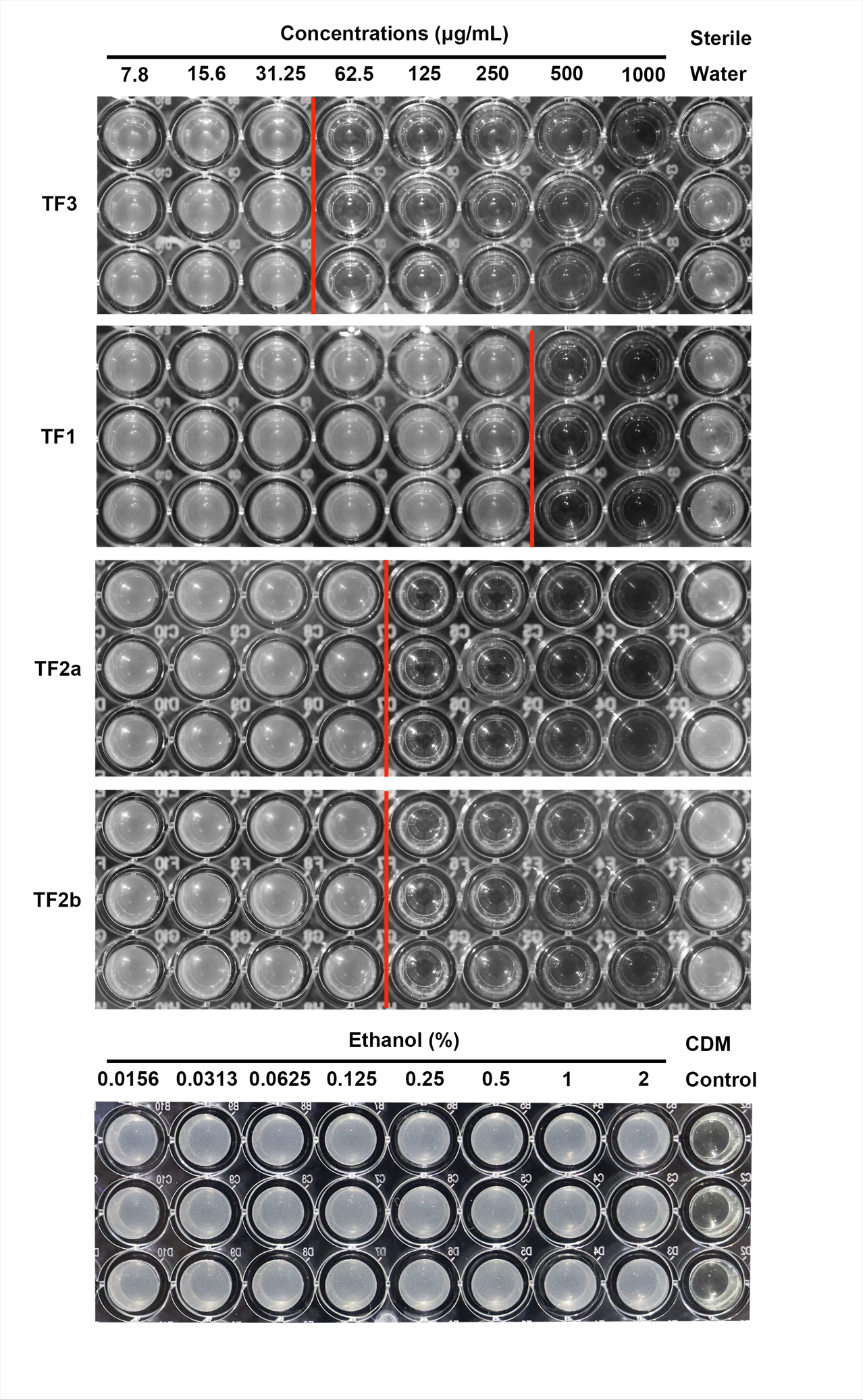


**Supplementary Figure 2**. *S. mutans* UA159 in CDM (5 × 10^6^ CFU/mL) was treated with four agents (TF3, TF1, TF2a, TF2b), ethanol (control) or sterile water (blank) in 96-well plates. The final concentrations of agents were 0 – 1000 μg/mL, and the ethanol concentrations in the control group were 0 – 2%. Pictures were taken at 24 h after the incubation. The MIC was defined as the minimum concentration of agent that inhibited the visible growth of the bacteria (TF3: 62.5 μg/mL, TF1: 500 μg/mL; TF2a: 125 μg/mL; TF2b: 125 μg/mL).


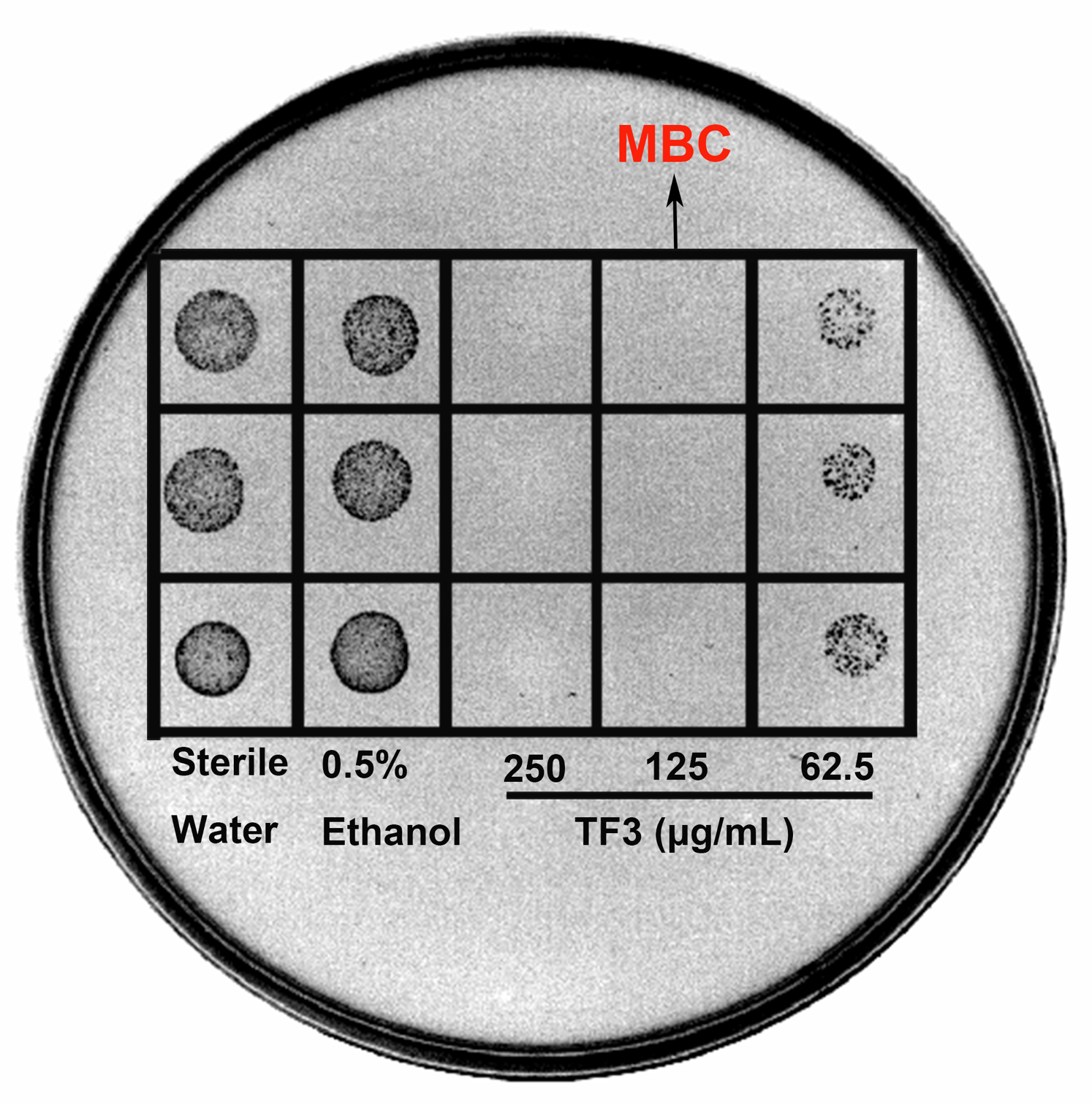


**Supplementary Figure 3**. Bacteria in cultures (plates from Figure S2) were collected by centrifugation, washed, re-suspended, and spotted onto THB agar plates (2 μL/dot). Pictures were taken at 48 h after the incubation. MBC was defined as the minimum concentration of TF3 that killed all the bacteria (125 μg/mL).


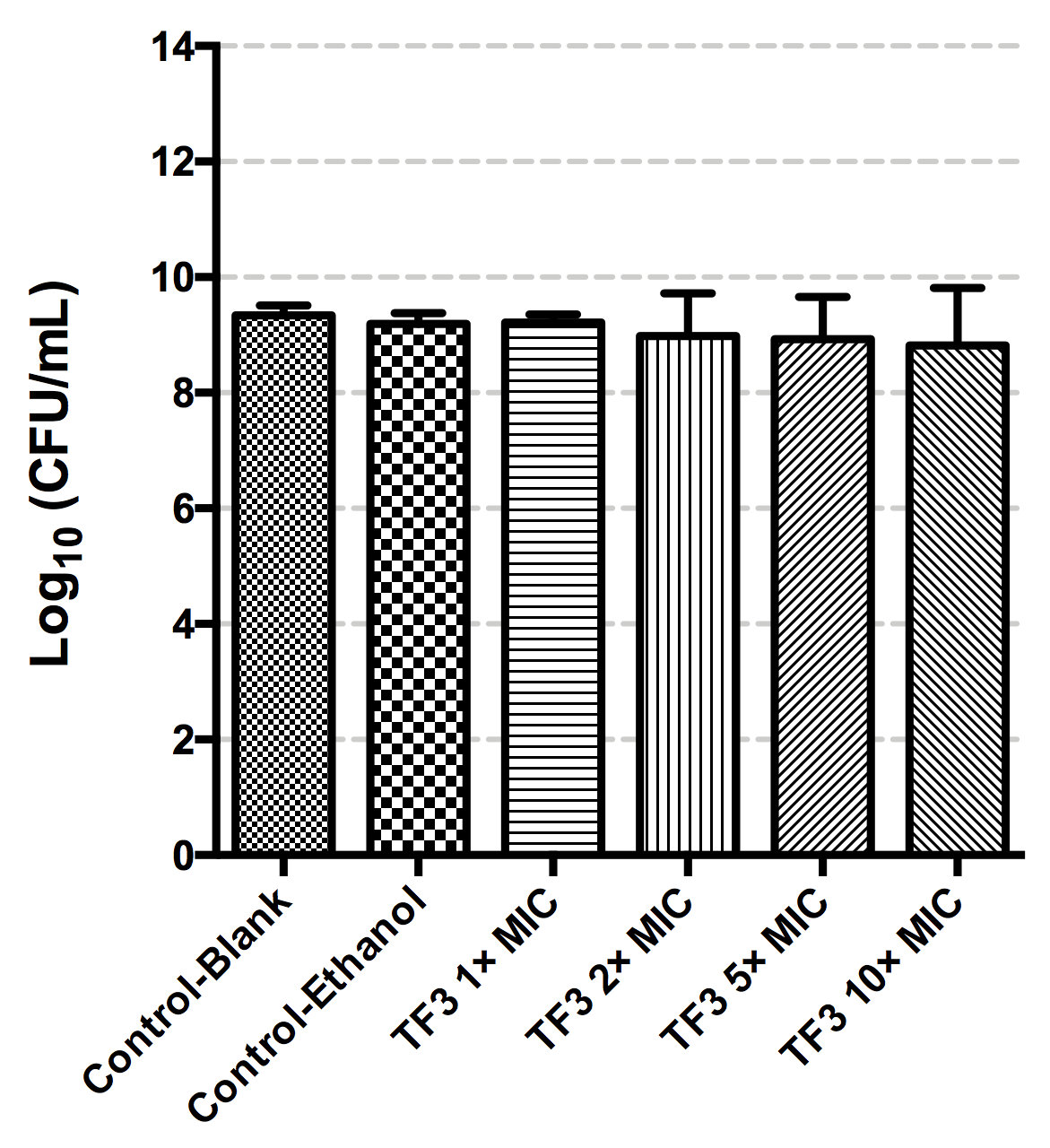


**Supplementary Figure 4**. Biofilms of *S. mutans* UA159 were preformed in CDMS in the wells of 96-well plates for 24 h, treated with 1× MIC, 2× MIC, 5× MIC and 10×MIC TF3 for 5min, washed with PBS, and resuspended in 200 μL PBS. Concentrations of living bacterial cells were determined by colony counting.


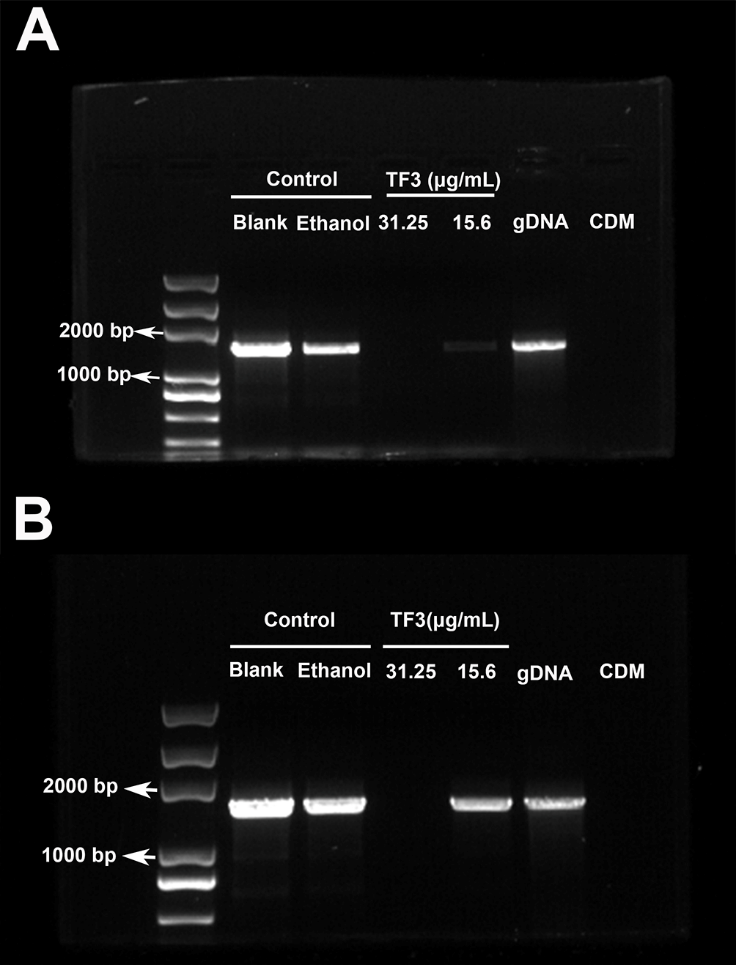


**Supplementary Figure 5**. The full scans of the entire original gels from the eDNA semi-quantification assays. Templates were collected at 6 h (A) or 24 h (B) after the incubation.

**Supplementary Table 1**. Concentrations of living bacterial cells (Log_10_ (CFU/mL)) of *S. mutans* UA159 treated with TF3 at different time points

| **Agents**  **Time (h)** | **Control** | | **TF3** | | | |
| --- | --- | --- | --- | --- | --- | --- |
|  | **Blank** | **Ethanol** | **1 × MIC** | **2 × MIC** | **5 × MIC** | **10 × MIC** |
| 1 | 6.61 ± 0.15 | 6.51 ± 0.09 | 6.62 ± 0.04 | 6.18 ± 0.03 | 6.01 ± 0.03 | 5.95 ± 0.04 |
| 3 | 6.76 ± 0.35 | 6.71 ± 0.24 | 6.46 ± 0.09 | 5.28 ± 0.27 | 4.09 ± 0.29 | 2.76 ± 0.33 |
| 6 | 8.19 ± 0.18 | 8.23 ± 0.11 | 6.31 ± 0.12 | 4.45 ± 0.38 | 2.63 ± 0.47 | 0 |
| 9 | 10.37 ± 0.04 | 10.18 ± 0.11 | 6.11 ± 0.11 | 4.45 ± 0.38 | 0.93 ± 1.31 | 0 |
| 24 | 10.18 ± 0.21 | 9.78 ± 0.24 | 4.07 ± 0.46 | 0 | 0 | 0 |

The data are shown as means ± standard deviations from three independent experiments.

**Supplementary Table 2**. Concentrations of living bacterial cells (Log_10_ (CFU/mL)) of *S. mutans* UA159 treated with sub-MIC TF3 for 24 h

|  | **Control** | | **TF3** | | |  |
| --- | --- | --- | --- | --- | --- | --- |
|  | **Blank** | **Ethanol** | **1/4 × MIC** | **1/2 × MIC** | **1 × MIC** | |
| Log_10_ (CFU/mL) | 9.37 ± 1.04 | 9.29 ± 0.91 | 8.81 ± 1.50 | 8.76 ± 1.59 | 4.05 ± 0.97^**^ | |

The data are shown as means ± standard deviations from three independent experiments. **, *P* < 0.01, when compared with the control-ethanol group using one-way ANOVA.

**Supplementary Table 3**. The effects of TF3 on biofilm formation of *S. mutans* UA159 in CDMS

|  | **Control** | | **TF3** | |
| --- | --- | --- | --- | --- |
|  | **Blank** | **Ethanol** | **1/4 × MIC** | **1/2 × MIC** |
| Biofilm formation (%) | 102.54 ± 11.74 | 100 | 26.80 ± 11.70^***^ | 10.10 ± 4.57^***^ |

The results are relative biomass of biofilms compared with the control-ethanol group. Data are shown as means ± standard deviations from three independent experiments. ***, *P* < 0.001, when compared with the control-ethanol group using one-way ANOVA.

**Supplementary Table 4**. The effects of TF3 on dextran formation of *S. mutans* UA159 in CDMS

|  | **Control** | | **TF3** | |
| --- | --- | --- | --- | --- |
|  | **Blank** | **Ethanol** | **1/4 × MIC** | **1/2 × MIC** |
| Dextran formation (%) | 98.71 ± 1.73 | 100 | 63.88 ± 2.64^***^ | 50.63 ± 3.90^***^ |

OD values of fluorescence excited by a Cascade Blue-conjugated dextran dye were measured and the results are relative dextran formation (OD values) compared with the control-ethanol group. Data are shown as means ± standard deviations from three independent experiments. ***, *P* < 0.001, when compared with the control-ethanol group using one-way ANOVA.

**Supplementary Table 5**. The effects of TF3 on eDNA formation of *S. mutans* UA159 in CDMS

|  | **Control** | | **TF3** | |
| --- | --- | --- | --- | --- |
|  | **Blank** | **Ethanol** | **1/4 × MIC** | **1/2 × MIC** |
| 6 h | 122.11 ± 48.89 | 100 | 15.33 ± 19.54^***^ | 0.89 ± 0.57^***^ |
| 24 h | 129.33 ± 47.55 | 100 | 21.56 ± 23.08^***^ | 3.44 ± 3.80^***^ |

PCR products were imaged using an ultraviolet camera after electrophoresis on 1% agarose gels and quantified using Image Lab. The results are relative eDNA production compared with the control-ethanol group. Data are shown as means ± standard deviations from three independent experiments. ***, *P* < 0.001, when compared with the control-ethanol group using one-way ANOVA.

**Supplementary Table 6**. The disperse effects of TF3 on preformed biofilms of *S. mutans* UA159

|  | **Control** | | **TF3** | | | |
| --- | --- | --- | --- | --- | --- | --- |
|  | **Blank** | **Ethanol** | **1 × MIC** | **2 × MIC** | **5 × MIC** | **10 × MIC** |
| Biofilm dispersion (%) | 121.87±21.29 | 100 | 96.95±11.89 | 102.73±18.24 | 109.52±17.52 | 118.88±18.06 |

The biofilm dispersion effects of TF3 on established *S. mutans* UA159 biofilms were detected by crystal violet staining and OD_595_ measurements. The results are relative biomass of biofilms compared with the control-ethanol group. Data are shown as means ± standard deviations from three independent experiments.

**Supplementary Table 7**. The bactericidal effects of TF3 on preformed biofilms of *S. mutans* UA159

|  | **Control** | | **TF3** | | | |
| --- | --- | --- | --- | --- | --- | --- |
|  | **Blank** | **Ethanol** | **1 × MIC** | **2 × MIC** | **5 × MIC** | **10 × MIC** |
| Log_10_ (CFU/mL) | 9.25 ± 0.18 | 9.21 ± 0.17 | 9.09 ± 0.30 | 8.36 ± 0.54^*^ | 3.22 ± 0.51^***^ | 0.60 ± 1.11^***^ |
| Relative concentrations of living cells (%) | 130.52 ±  76.36 | 100 | 105.46 ±  69.17 | 28.36 ±  28.04^**^ | 0.00024 ±  0.00040^***^ | 0.0000075 ±  0.000028^***^ |

The bactericidal efficiency of TF3 at different concentrations against established *S. mutans* UA159 biofilms was determined by colony counting. The results are shown as relative concentrations of living cells compared with the control-ethanol group. Data are shown as means ± standard deviations from three independent experiments. *, *P* < 0.05; **, *P* < 0.01 and ***, *P* < 0.001, when compared with the control-ethanol group using using one-way ANOVA.

**Supplementary Table 8**. The pH of *S. mutans* UA159 supernatants in salt solutions with 1% glucose.

| **Time (min)**  **Agents** | **Control-Blank** | **Control-Ethanol** | **TF3 1× MIC** |
| --- | --- | --- | --- |
| 0 | 7.2 | 7.2 | 7.2 |
| 10 | 6.83 ± 0.087 | 6.76 ± 0.086 | 7.05 ± 0.048 |
| 20 | 6.37 ± 0.034 | 6.31 ± 0.15 | 6.92 ± 0.083 |
| 30 | 5.97 ± 0.042 | 5.96 ± 0.15 | 6.77 ± 0.085 |
| 40 | 5.64 ± 0.070 | 5.53 ± 0.24 | 6.58 ± 0.11 |
| 60 | 5.17 ± 0.015 | 5.12 ± 0.28 | 6.15 ± 0.21 |
| 80 | 4.83 ± 0.068 | 4.71 ± 0.25 | 5.71 ± 0.21 |
| 100 | 4.38 ± 0.037 | 4.44 ± 0.27 | 5.48 ± 0.18 |
| 120 | 4.19 ± 0.10 | 4.30 ± 0.23 | 5.32 ± 0.096 |
| 360 | 3.93 ± 0.054 | 3.90 ± 0.059 | 4.60 ± 0.11 |

Data are shown as means ± standard deviations from three independent experiments.

**Supplementary Table 9**. The relative concentrations of living cells in biofilms of *S. mutans* UA159 treated with TF3 and acid.

| **pH**  **Agents** | **Control-Blank** | **Control-Ethanol** | **TF3** **1× MIC** |
| --- | --- | --- | --- |
| 7 | 108.37 ± 11.69 | 100 | 94.94 ± 4.31 |
| 1 | 98.09 ± 36.34 | 100 | 17.60 ± 3.68^***^ |
| 1.25 | 125.72 ± 12.12 | 100 | 9.76 ± 5.23^***^ |
| 1.5 | 109.05 ± 10.72 | 100 | 2.90 ± 1.84^***^ |
| 1.75 | 111.90 ± 5.41 | 100 | 13.14±6.49^***^ |
| 2 | 123.40 ± 6.89 | 100 | 56.98 ± 31.00^**^ |

The results are shown as relative concentrations of living cells compared with the control-ethanol group. Data are shown as means ± standard deviations from three independent experiments. ***, *P* < 0.001, when compared with the control-ethanol group using using two-way ANOVA.

**Supplementary Table 10**. The relative expression levels (2 ^-△△CT^) of genes in *S. mutans* UA159 during biofilm formation

| **gene**  **Agents** | **Control** | | **TF3** | |
| --- | --- | --- | --- | --- |
|  | **Blank** | **Ethanol** | **1/4 × MIC** | **1/2 × MIC** |
| *gtfB* | 1.10 ± 0.41 | 1 | 0.067 ± 0.044^***^ | 0.025 ± 0.012^***^ |
| *gtfC* | 1.06 ± 0.49 | 1 | 0.0014 ± 0.00097^***^ | 0.0010±0.00064^***^ |
| *gtfD* | 1.16 ± 0.61 | 1 | 0.0070 ± 0.0089^***^ | 0.0013±0.00079^***^ |
| *lrgA* | 0.88 ± 0.31 | 1 | 0.046 ± 0.017^***^ | 0.018 ± 0.013^***^ |
| *lrgB* | 0.87 ± 0.27 | 1 | 0.20 ± 0.10^***^ | 0.057 ± 0.042^***^ |
| *lytS* | 0.88 ± 0.19 | 1 | 0.073 ± 0.075^***^ | 0.012 ± 0.0041^***^ |
| *lytT* | 1.21 ± 0.45 | 1 | 0.039 ± 0.017^***^ | 0.027 ± 0.0075^***^ |
| *srtA* | 1.21 ± 0.31 | 1 | 0.011 ± 0.0079^***^ | 0.0068 ± 0.0051^***^ |
| *vicK* | 0.84 ± 0.27 | 1 | 0.023 ± 0.028^***^ | 0.0016 ± 0.0016^***^ |
| *vicR* | 0.92 ± 0.16 | 1 | 0.048 ± 0.049^***^ | 0.0075 ± 0.012^***^ |
| *comD* | 0.99 ± 0.20 | 1 | 0.040 ± 0.036^***^ | 0.034 ± 0.025^***^ |
| *comE* | 0.72 ± 0.45 | 1 | 0.045 ± 0.077^***^ | 0.0072 ± 0.0059^***^ |

Data are shown as means ± standard deviations from three independent experiments. ***, *P* < 0.001, when compared with the control-ethanol group using using one-way ANOVA.

**Supplementary Table 11**. The relative expression levels of genes in *S. mutans* UA159 bacterial cells in preformed biofilms

| **gene**  **Agents** | **Control** | | **TF3** |
| --- | --- | --- | --- |
|  | **Blank** | **Ethanol** | **1 × MIC** |
| *ldh* | 0.84 ± 0.21 | 1 | 0.042 ± 0.032^***^ |
| *eno* | 0.93 ± 0.57 | 1 | 0.039 ± 0.027^***^ |
| *atpD* | 1.01 ± 0.60 | 1 | 0.074 ± 0.032^***^ |
| *aguD* | 0.69 ± 0.33 | 1 | 0.017 ± 0.019^***^ |
| *vicK* | 1.01 ± 0.40 | 1 | 0.014 ± 0.013^***^ |
| *vicR* | 0.95 ± 0.61 | 1 | 0.12 ± 0.11^***^ |
| *comD* | 1.21 ± 0.33 | 1 | 0.11 ± 0.10^***^ |
| *comE* | 0.80 ± 0.46 | 1 | 0.089 ± 0.11^***^ |

Data are shown as means ± standard deviations from three independent experiments. ***, *P* < 0.001, when compared with the control-ethanol group using using one-way ANOVA.
